# Challenges and Opportunities of System-Level Prognostics

**DOI:** 10.3390/s21227655

**Published:** 2021-11-18

**Authors:** Seokgoo Kim, Joo-Ho Choi, Nam H. Kim

**Affiliations:** 1 Department of Mechanical and Aerospace Engineering, University of Florida, Gainesville, FL 32611, USA; seokgoo.kim@ufl.edu; 2 School of Aerospace and Mechanical Engineering, Korea Aerospace University, Goyang 10540, Korea; jhchoi@kau.ac.kr

**Keywords:** system-level prognostics, performance, remaining useful life, dependency, multiple components, challenges

## Abstract

Prognostics and health management (PHM) has become an essential function for safe system operation and scheduling economic maintenance. To date, there has been much research and publications on component-level prognostics. In practice, however, most industrial systems consist of multiple components that are interlinked. This paper aims to provide a review of approaches for system-level prognostics. To achieve this goal, the approaches are grouped into four categories: health index-based, component RUL-based, influenced component-based, and multiple failure mode-based prognostics. Issues of each approach are presented in terms of the target systems and employed algorithms. Two examples of PHM datasets are used to demonstrate how the system-level prognostics should be conducted. Challenges for practical system-level prognostics are also addressed.

## 1. Introduction

**Figure 1:**
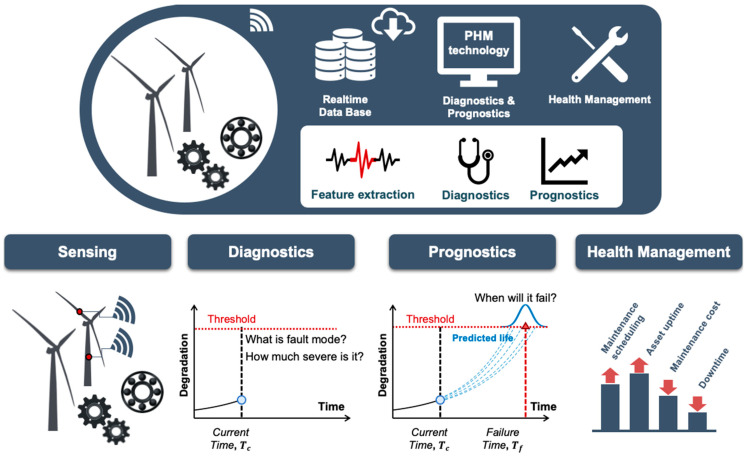
Levels of prognostics and health management.

Condition-based maintenance (CBM) is a maintenance policy that maintains the reliability of system operation and reduces the downtime of the system. Prognostics and health management (PHM) has attracted much attention as the enabler of CBM. The PHM aims to predict the remaining useful life (RUL) of the system and suggest an optimal health management strategy. The PHM consists of four main stages: sensing, diagnostics, prognostics, and health management, which are illustrated in Figure 1. In the sensing stage, PHM engineers determine what to measure and which kind of sensors to install. Health diagnostics is the process of evaluating the degree of damage significance and identifying the root causes of failure. In other words, it focuses on the current operability of the system at stake. On the other hand, health prognostics aims to provide information about the future operability of the system. Prognostics includes establishing a failure precursor which indicates an incipient degradation of the system and estimates the RUL based on the current health state and expected future operating conditions [1]. Finally, the health management of the system is performed based on the information obtained from diagnostics and prognostics. Each step has its own challenges. For example, effective sensor network design for sensing [2], feature extraction, observability analysis, and diagnostics algorithm for fault diagnostics [3,4,5], development of prognostics algorithm [6], and proper system operation strategy for health management [7]. In view of the CBM, however, the prognostics is the most important since it enables the proactive maintenance plan [1,8]. This article focuses on the prognostics of complex systems that are encountered in the real industry.

To date, there are many valuable review papers and books in the PHM with diverse aspects such as the general process of PHM [1,9,10,11,12,13,14,15], pre-processing [16,17], and prognostics algorithms [18,19,20,21,22]. For example, Lee et al. [1] provided a comprehensive review of the PHM followed by an introduction of a systematic PHM design methodology for converting data into prognostic information. Lei et al. [14] provided a systematic review of machinery prognostics from the data acquisition to the RUL prediction and summarized several prognostics datasets commonly used for the research. An et al. [22] presented practical options for prognostics to select an appropriate method for different applications. All the reviews have provided successful case studies and useful descriptions of prognostics algorithms. However, most of the reviews have focused on the component-level prognostics, such as the bearings [23,24], gears [25,26], and batteries [27,28,29].

**Figure 2:**
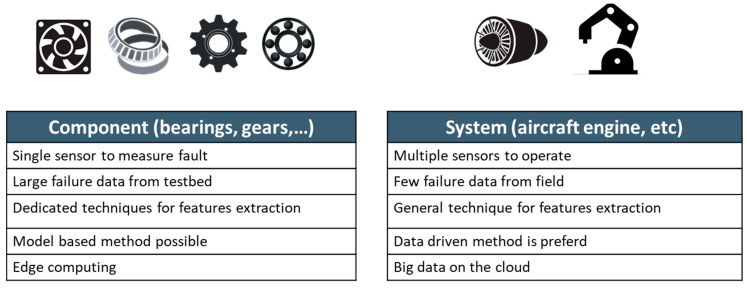
Prognostics approach for component and system level.

As the industrial systems in the field become more complex, comprising of multiple components, system-level prognostics is gaining much more interest from industry and academia. A complex system is composed of many interlinked components, which makes the system-level prognostics difficult [10,30]. It should be noted that the degradation and health condition of the system is determined by its components, which means that the individual degradation of components should be explored first and integrated to assess the system performance [10,31]. From the research viewpoint, the system-level prognostics has different characteristics from those of the component-level as summarized in Figure 2. At the component level, a single or a set of sensors, such as vibration, acoustic emission, and temperature sensors, can be used to monitor damage degradation. Since components are relatively easy to test, a large number of failure data can be obtained from a testbed for the algorithm development. In addition, a dedicated algorithm can be developed for feature extraction of the target component. On the contrary, system-level prognostics contains multiple sensors from various components. Dedicated algorithms may not work in one way or the other in the system. Models are rarely available due to the system complexity, which means that the data-driven method may be the only option. Few or no failure data exist in the real operation or by the testbed. All these are the issues around the system-level prognostics.

Despite its importance and challenges, only a few reviews are found on the system-level prognostics [30,32]. Li et al. [32] summarized prognostics algorithms for rotating machinery. Bektas et al. [30] reviewed prognostics algorithms and RUL estimation of complex systems under multiple operating regimes. However, these reviews have been limited to the prognostics algorithms while missing the other more important issues in the system-level prognostics. In fact, the algorithms in the review are not just limited to the systems but are valid in the general sense. In light of this, the aim of this paper is to review the current issues of system-level prognostics, survey how they have been addressed in the literature, and suggest future challenges toward the practical applications. To achieve this goal, the scope and definition of system-level prognostics in this paper are specified by the following three points: First, the testing-based means the prognostics using the event data, which are the end of life collected from the past operations or the reliability tests. The life is estimated by statistical methods from the failure data. The reason to mention this is because a considerable number of papers have addressed this for the system-level prognostics, which is the population-based approach, hence, is not the scope of this paper. Second, since the PHM is more concerned with the individual health of the system, the survey is limited to the condition-based, which addresses the condition monitoring (CM) data of individual systems [33,34]. As mentioned before, the true prognostics is about the RUL prediction based on the health estimation so far. Upon the survey, however, it was found that many have remained with the health index development of the system. In this sense, the scope covers this as long as it deals with the system health inter-connected with the components. Third, the scope addresses the issue of multiple failure modes, which may occur in a single component. But it is treated as the topic of system-level prognostics as well. The abovementioned three points can be summarized as follows:

- Condition-based prognostics, not testing-based prognostics
- Health index development for multiple component systems
- Prognostics of multiple failure modes

Under this background, this paper has surveyed literature, and categorized them into four approaches: system health-index based, integration of components’ RUL into the system, prognostics under influenced components, and prognostics of multiple failure modes. The first focuses on health index development. The second deals with how the components’ RUL are integrated into the system. The third handles the interdependency between components. The fourth is for the multiple failure modes.

The paper is organized as follows: brief reviews on the prognostics algorithms for system-level prognostics are provided in Section 2. In Section 3, four approaches for the system-level prognostics are explained along with their issues. In Section 4, existing datasets for system-level prognostics are introduced. Challenges for effective system-level prognostics are presented in Section 5, followed by conclusions in Section 6.

## 2. Algorithms for System-Level Prognostics

While there have been similar papers that have reviewed algorithms for the system-level prognostics [32], this section summarizes them once again very briefly for the purpose of integrity as they appear in the subsequent sections. It is again emphasized that the algorithms reviewed herein are not limited to the system. In general, prognostics algorithms are categorized into physics-based and data-driven approaches [10]. Although some literature mentions a third category such as knowledge-based or hybrid approach, this paper classifies them into the data-driven because most literature does so. Physics-based methods describe the evolution of damage using comprehensive mathematical models based on the physics-of-failure and degradation of system performance. Commonly used ones are the Paris model [35] and Huang model [36] in the case of fatigue crack growth. They are usually combined with Particle filter (PF) [37] or Kalman filter (KF) [38] in order to estimate the RUL before the crack reaches a critical size. In the case of complex systems, however, physics-based approaches are not likely applicable due to the complexity of systems inter-connected by multiple components. Data-driven approaches utilize the CM data collected from the installed sensors and build mathematical models for the RUL estimation. As it does not require domain or physical knowledge, many publications have focused on this approach [39,40,41,42]. The performance of data-driven prognostics, however, heavily depends on the number and quality of data, as it requires a large number of trend or run-to-failure data for accurate model construction. Up to date, various types of prognostics algorithms have been introduced such as PF [43,44,45], neural network [46,47], support vector machine [48], survival analysis [49], and Cox’s proportional hazard model [50,51]. Among them, several algorithms that have been used for system-level prognostics are briefly introduced in the following section.

### 2.1. Particle Filter

Whether it is component or system, as long as a degradation model and CM data are available, a physics-based approach can be used, which estimates the model parameters representing the system health based on the CM data. As a means to this end, the particle filter (PF) algorithm is the most commonly used [31,45,52], which is rooted in the Bayesian inference as follows

**Figure 3:**
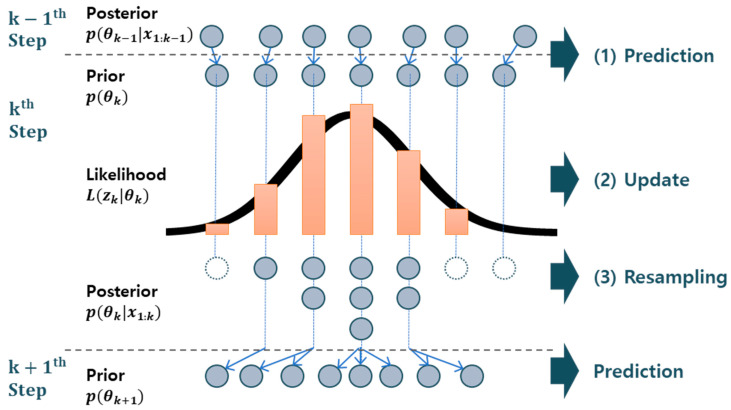
Illustration of the particle filter process.

(1) p(θ|x)∝L(x|θ)p(θ)

where θ and *x* represent the vector of unknown parameters and observation data, respectively. The posterior distribution p(θ|x) is proportional to the multiplication of the prior distribution and the likelihood function, which are expressed as p(θ) and L(x|θ), respectively. The particle filter represents the distribution via a large number of particles. It consists of two equations: recursive representation of the degradation model and the measurement equation. The process is composed of three steps: (1) prediction of the parameters at the current time step *k* from the previous *k*-1 using the recursive equation; (2) update of the parameters using the likelihood function based on the measured data at the current time; and (3) resampling of the updated particles based on their weights derived from the likelihood. The resampled particles are used as a prior distribution at the next time step. The process is illustrated in Figure 3.

### 2.2. Artificial Neural Network

**Figure 4:**
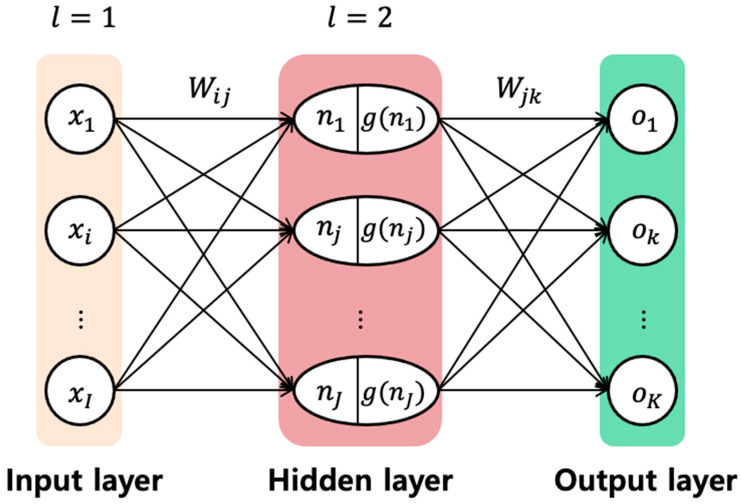
Architecture of an artificial neural network.

When there is no model available, the artificial neural network (ANN) is the viable option, which is widely used for data-driven prognostics. It aims to map input data such as various sensor signals and their time histories into the output data such as the health degradation or the RUL. The ANN architecture contains three layers: input layer, single or more hidden layers, and output layer, as shown in Figure 4. Each layer contains neurons (nodes) and weights that are illustrated as circles and arrows, respectively. The input nodes $x_{i}\;\left(i=1,\ldots ,I\right)$ are multiplied by weights $W_{ij}$ to obtain the values $n_{j}$, which become the input to the activation function *g* at the hidden layer [53]. The same process is performed when a hidden node *j* is mapped into the output node $o_{k}$. Given the input and output data, the ANN is trained to determine the optimum weights such that the network describes closely the relationship between the input and output. To further improve the accuracy, optimum number of hidden layers and nodes are determined as well via cross validation. As an advanced ANN, recurrent neural network (RNN) [54], convolutional neural network (CNN) [55], and long short term memory (LSTM) [56] have been widely used for prognostics recently.

### 2.3. Similarity-Based Method

**Figure 5:**
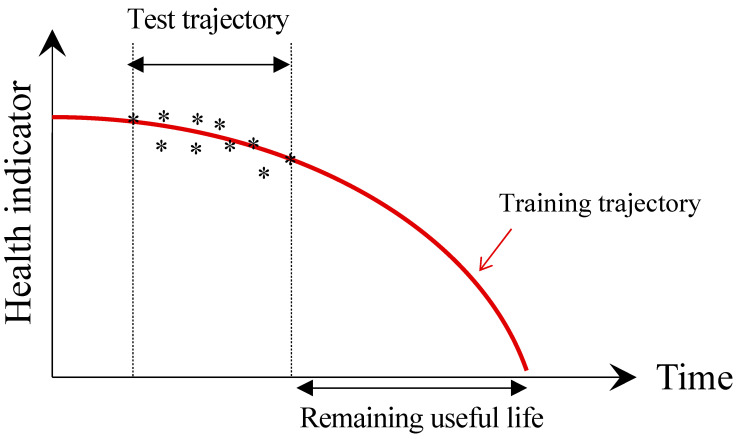
Similarity-based RUL prediction. Asterisk markers represent the test trajectory.

When a large number of run-to-failure data are available from the past operation, a similarity-based RUL prediction method can be applied [57]. The method evaluates the similarity between the current test data (to predict the RUL) and the past training data (obtained until failure) to identify the best matching portion of the degradation trend and use it for the RUL prediction of the current system. The RUL is estimated by the past RULs of training datasets, which are weighted based on the degree of similarity. This is quite a unique approach, distinct from the extrapolation methods like PF or ANN-based training [58,59]. Figure 5 illustrates the similarity-based method, which indicates that when the current health index data are located along the past training trajectory as shown in the figure, the highest similarity is achieved. Then the RUL is determined by the past trajectory from the end of current data. The similarity is evaluated by the distance between two trajectories, given by [47]

(2) $$d_{\left(tr,te\right)}=\sqrt{\sum_{i=1}^{n}{\left(te_{i}-tr_{i}\right)}^{2}}$$

where *te* and *tr* represent the test trajectory and the corresponding training trajectory, respectively, and *n* is the length of the test trajectory. As its prognostics performance depends on the similarity evaluation, several references focus on establishing effective similarity measures and quantification of uncertainty [47,60,61].

### 2.4. Cox Proportional Hazard Model

Over the past years, the Cox proportional hazard model has been developed, which is quite different from the previous algorithms. While the former considers the RUL prediction of individual assets using the CM data, the Cox model does this on a population basis using the statistical analysis, but accounts for the severity of degradation using the CM data. In fact, the model predicts the hazard (or failure rate) of a system by combining the historical failure data and online CM data [62]. In the model, the CM data, often called covariates, are used to reflect the severity from the baseline hazard rate. Then the hazard model, which represents the failure rate undergoing the conditions featured by the CM data, is defined as follows.

(3) $$\lambda \left(t\right)=\exp\left(z^{T}\beta \right)\lambda_{0}\left(t\right)$$

where λ(t) represents the hazard rate at time *t*, $\lambda_{0}\left(t\right)$ is the baseline rate without the influence of covariates determined by the system lifetime data. z and **β** are the CM data and the corresponding vector of unknown parameters to be estimated by the maximum likelihood using the failure times and CM data [62,63].

## 3. Approach for System-Level Prognostics

Based on the issues and challenges mentioned in the introduction, this section reviews the approaches that have been addressed to solve the system-level prognostics. It can be grouped into four categories: (1) system health index-based, (2) integration of components’ RUL, (3) prognostics under influenced components, and (4) multiple failure modes. To help readers understand, authors have added simple illustrative examples in each category. It should be noticed that each approach is not about a specific prognostics algorithm but the way to integrate the information from multiple components for system-level information. In this paper, this process is called ‘systematization’. Therefore, any prognostics algorithms can be used before performing the systematization.

### 3.1. Approach 1: System Health Index-Based Approach

In the system health index-based approach, the health index is introduced to represent the degradation state of the system. Ideally speaking, the system health index should be derived from the degradation of each component. This is however hard to achieve because the relationship between the components and system is usually unknown. Under this circumstance, the system health index-based method can be further divided into three groups: (1) physical system performance (PSP)—physical outputs such as the flow rate of a piping system or the generated power of wind turbine as an example, (2) virtual system performance (VSP)—index representing the system health such as the probability of system failure or distance from the normal; and (3) direct RUL of the system. Among the three groups, the PSP, which employs a physical model, has a strength in both physical interpretation and prediction accuracy. However, such a model is rarely available for complex systems. Thus, the VSP and direct RUL are taken as more practical options, which is also challenging since a large number of run-to-failure data are required.

**Figure 6:**
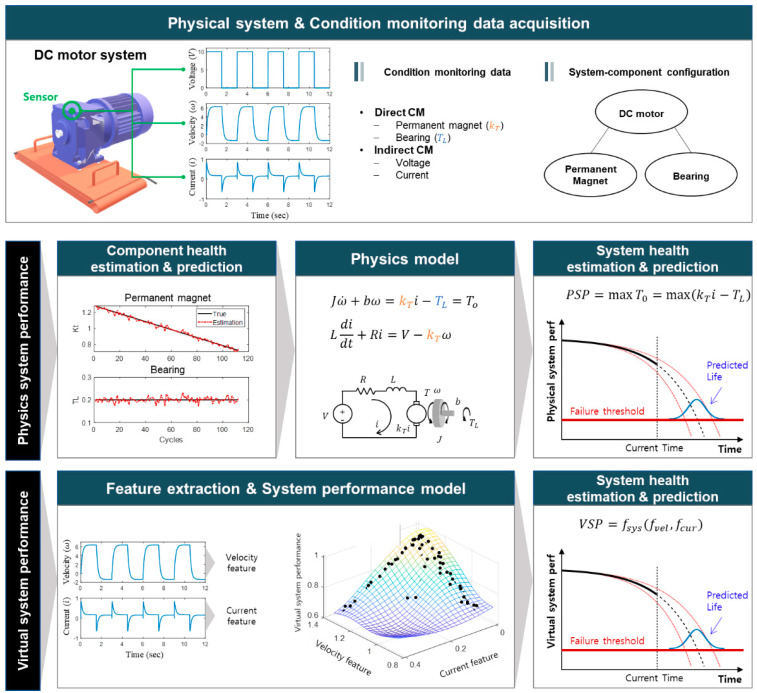
System health index-based approach.

Figure 6 shows the example of a DC motor to aid in explaining the system health index-based method. It should not be confused that the motor here is regarded as a system consisting of two components: permanent magnet and bearing, whose degradation affects the system performance: the reduction in the output torque of the motor. Typically, the velocity and current are obtained as the CM data. In the PSP method, system health (e.g., the output torque of DC motor, $T_{O}$) is estimated via a physical system model, in which the degradation of the components and the resulting system health are evaluated based on the CM data. In the VSP method, virtual system health is commonly introduced between 1 (normal) and 0 (failure) or vice versa, and an empirical model is developed to relate the CM data with the system health using the run-to-failure data set. For this, a machine learning algorithm whose inputs are features extracted from signals and output is health index between 0 and 1 is usually employed.

While the overall summaries for each approach in the literature are given in Table 1, a few papers are explained in more detail. In the PSP approach, Rodrigues [64] estimated system RUL using the system-level performance indicator obtained by the system model. He converted the health factors of individual components into the performance indices and combined them into the system-level performance. Khorasgani et al. [31] developed a two-step process for the system prognosis. In the estimation step, the system state and degradation parameters are estimated based on the system model using the PF. Then in the prediction step, the first-order reliability method (FORM) is applied to predict the system RUL. In their work, the system EOL was defined based on the system performance, which was calculated from the individual components and system degradation model. Wang et al. [65] introduced a Bayesian network-based lifetime prediction method for systems, which combines multiple sensor information and considers the interdependency between accidental failure and degradation failure mechanism. Liu et al. [66] developed a dynamic reliability assessment approach for the multi-state system by utilizing the system-level observation history. The proposed recursive method dynamically updates the reliability function of the system by incorporating system-level inspection data.

In the VSP approach, a virtual system health index is mainly introduced that varies between 1 in the early period and 0 near the failure. Then, logistics regression [67] or linear regression [68] are used as an empirical system model to convert the CM data into 1D system performance. The elevator door [67] or aircraft engine [68] are chosen for the demonstration. Other researchers have employed the concept of distance from the normal as the health indicator, which is determined by multivariable state estimation technique (MSET) [69], auto-associative kernel regression (AAKR), or auto-associative neural networks (AANN) [70,71]. The direct RUL method is similar to the VSP but the RUL is employed directly instead of the VSP. That is, the CM data are directly related with the RUL of target assets using artificial intelligence (AI) algorithms, such as multi-layer perceptron (MLP) [72,73], convolutional neural network (CNN) [74,75], recurrent neural network (RNN) [40,76], and long short-term memory (LSTM) [42,46,56], in which the system-model is considered as a black-box. There have also been studies in which the health index is first developed for the system, and the RUL prediction by the index is followed using such as the particle filter [52], the similarity-based method [47,58,68], and the ensemble approach [77]. It should be remarked that although these papers address the system in their study, it is not strictly the system prognosis since they treat the system as a single unit without considering the components.

### 3.2. Approach 2: Integration of Components’ RUL into the System

**Figure 7:**
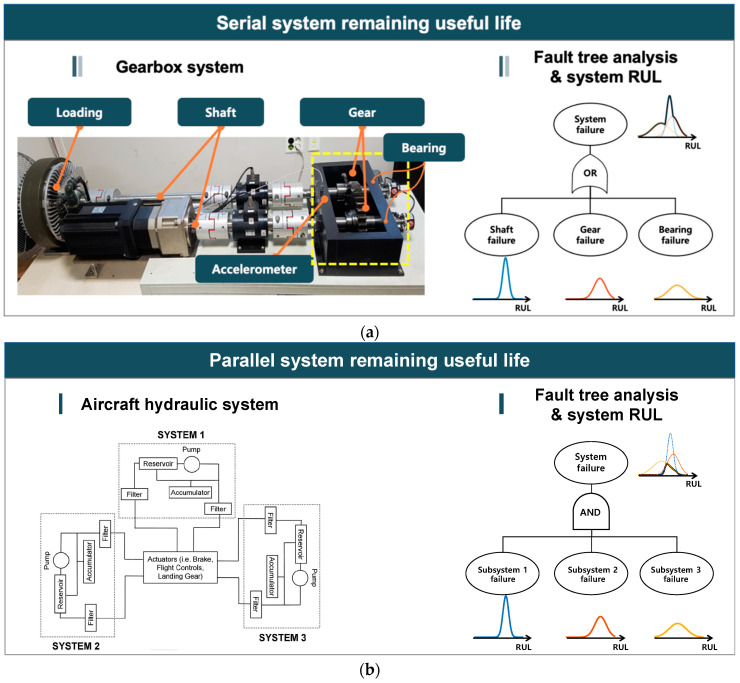
Component RUL-based approach: (**a**) System RUL of serial systems (gearbox system); (**b**) System RUL of parallel systems (aircraft hydraulic system).

The second approach is to integrate RUL information of individual components to obtain the system-level RUL, rather than directly determining the system health index or RUL as in approach 1. Figure 7 briefly illustrates the component RUL-based approach. In the figure, two examples of the serial and parallel system are given, which define the system failure based on the ‘AND’ and ‘OR’ gates of the fault tree diagram. For the gearbox system in Figure 7a, failure of any components results in system failure. In this case, the union of three RULs yields the system RUL. For the aircraft hydraulic system with redundancy, the failure of all three sub-systems leads to system failure as shown in Figure 7b, which means that the intersection of three RULs gives the system RUL.

The diagram can be generalized to the complex system by applying the fault tree analysis (FTA), in which the component-level RULs are propagated to the system RUL by the fault tree structure (see, e.g., Gomes et al. [85]). Ferri et al. [86] proposed a methodology for maintenance planning in the view of system-level prognostics using the FTA. In the end, the system-level RUL was used to identify optimum component combinations to be repaired in order to maximize system safety. In this category, some literature has employed a physical system model to determine the RUL of individual components. This approach, however, results in a higher computational burden as the number of components increases. To overcome this issue, model decomposition methods have been proposed by Daigle et al. [87,88,89], in which a distributed approach is developed for the system-level prognostics by decomposing both the estimation and prediction problems into computationally independent sub-scale problems. Then the system RUL is determined as a minimum of the independent subsystem’s RUL. They have also developed PF-based prognostics characterizing multiple damage progression paths based on the joint state-parameter estimation [90]. Vasan et al. [91] proposed approaches based on decomposing the system into multiple critical circuits and exploiting the parameters specific to the system’s circuits. Chiachio et al. [92] introduced a mathematical framework for modeling prognostics at a system level based on the plausible Petri net by incorporating maintenance actions, various prognostics information, expert knowledge and resource availability. Table 2 summarizes the component RUL-based methods for system-level prognostics.

### 3.3. Approach 3: Prognostics under Influenced Components

**Figure 8:**
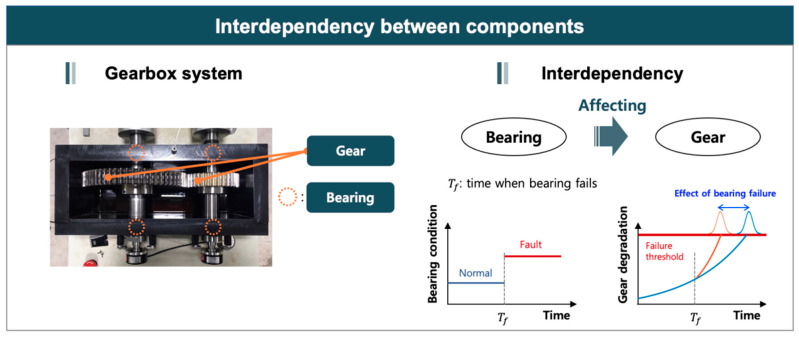
Influenced component-based approach.

As mentioned before, system-level prognostics is difficult due to the inter-dependencies between the “affecting” and “influenced” components in the system [10,31]. Such dependencies may lead to the different degradation of the system than the case otherwise. Figure 8 shows the gearbox system, which consists of gear and bearing, where the degradation or fault of bearing affects the degradation of gear. In the figure, if the bearing stays in the normal condition, the health trend of gear shows the normal degradation pattern. When a fault occurs in the bearing, however, the degradation pattern of gear is changed, i.e., is accelerated, and reaches the threshold earlier. This issue has already been studied extensively in the field of maintenance strategies and policies with the topic of the multiple components [96]. However, they did not consider the interdependency of the components in the prognostics or RUL prediction.

While the list of papers for this approach is given in Table 3, some of them are explained in detail as follows. Tamssaouet et al. [97,98,99,100,101,102] proposed a methodology based on the inoperability input-output model to evaluate the system-level RUL in the situation where multiple interactions between components and the influence of the environment exist. Liu et al. [103] introduced dynamic reliability assessment and RUL prediction of a system that consists of a pump and valve. Parallel Monte Carlo simulation and recursive Bayesian method are integrated for the purpose of failure prognostics under dependency among components. Hu et al. [104] proposed a failure prognosis method using the dynamic Bayesian network (DBN) for a complex system, which considers the interaction between components and influence of protection action in the system during dynamic failure scenarios. Maitre et al. [105] emphasized that when one component has a failure, the remaining components compensate for the loss of the component and thus function in a ‘boosted’ mode. As a result, the component under ‘boosted’ mode shows a more severe degradation than without it. Hafsa et al. [106] emphasized the importance of interactions between components in RUL prediction. They proposed a method combining the probabilistic Weibull and stochastic dependency model, which characterizes the effects of degradation interaction derived from other components. Hanwen et al. [107] demonstrated that there exists a noise that impacts the system with multiple components, as all the components operate in the same circumstance and affect each other. They named this public noise. To describe the degradation with public noise, Brownian motion that affects the degradation of components was added to the Wiener process. Then, the degradations of the components are jointly estimated by the KF, and the system RUL is determined by the minimum RUL of components. Bian and Gebraeel [108,109] proposed a stochastic modeling methodology considering interactions among the degradation of components in a system. They focused on characterizing the relationship between the influencing and the affected component.

### 3.4. Approach 4: Prognostics of Multiple Failure Modes

**Figure 9:**
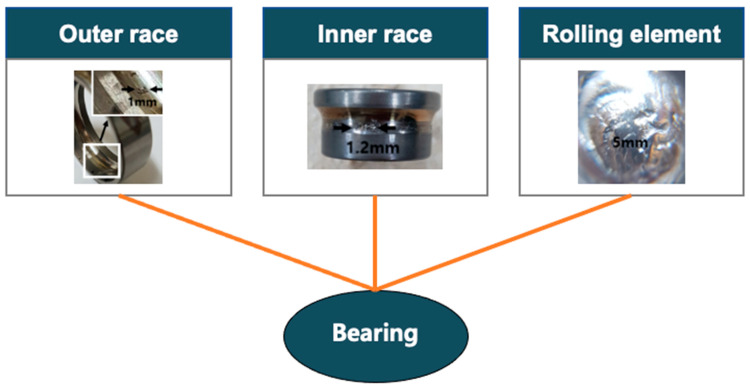
Illustration of failure mode-based approach.

In the PHM, identification of fault modes is the initial step toward successful prognostics [58]. In many cases, the system contains multiple failure modes even for a single component. In that case, the degradation of components or systems can show a different pattern from those of single mode, which should involve identifying active failure modes and tracking their progression. The case is illustrated by an example in Figure 9, where the bearing faults can occur at different places with different progression paths such as the outer race, inner race, and rolling element. The faults if occurred concurrently can interact and accelerate the global degradation of the components [90].

For accurate fault prognosis, the method should be able to address this aspect. Several approaches have been studied to this end, most of which were however rooted in the traditional reliability engineering such as a hazard model or survival analysis [116,117,118,119]. Ragab et al. [116] merged the logical analysis of data with a set of non-parametric cause-specific survival functions and applied it to the bearing prognostics whose failure modes were inner race, outer race, and rolling element faults. Zhang et al. [118] presented a mixture Weibull proportional hazard model for the EOL estimation of mechanical system that includes multiple failure modes and applied to a pump system that contains two failure modes: sealing ring wear and thrust bearing damage. Historical lifetime and condition monitoring data were combined into the traditional proportional hazard model. Blancke et al. [120] introduced a multi-failure mode prognosis approach for complex equipment. They used graph theory and stochastic models for diagnostics and prognostics, respectively. Once the failure mechanism is detected by the diagnostic process, the prognostic algorithm based on a stochastic model is used to predict the possible failure mode dynamically as new data are acquired. The proposed algorithm was applied to a hydroelectric generator stator, which contains more than 150 failure mechanisms associated with three failure modes. While the above studies are based on the traditional reliability approach, there have been other studies for the multiple failure modes prognosis by using the PF [90,121,122,123]. Daigle and Goebel [123] used the PF for model-based prognostics of a valve system that contains multiple failure modes. Zhang et al. [121] introduced PF-based multi-fault prognostics of bearing degradation whose failure modes were grease damage, spall, and unknown fault. They monitored features directly related to each failure mode and utilized them in the PF framework. Table 4 summarizes the system-level prognostics considering multiple failure modes.

## 4. Datasets for System-Level Prognostics

So far, there have been many run-to-failure datasets published from several institutions such as NASA Ames, FEMTO, and PHM society. However, most of the existing datasets were associated with component-level problems such as bearing, battery, and filter clogging. The main challenge in system-level prognostics research is the lack of available datasets. So far, only two datasets are open to the public, to the authors’ knowledge. This section summarizes these datasets and suggests which approaches are good to answer the questions of the problem in view of system-level prognostics.

### 4.1. C-MAPSS Datasets

**Figure 10:**
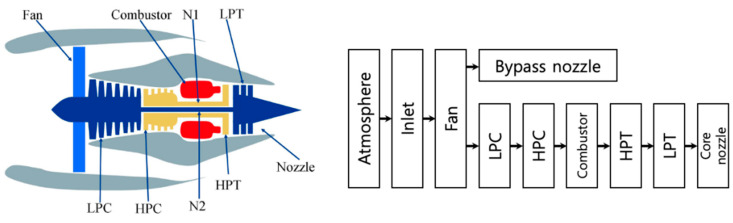
Diagram of the simulation model of C-MAPSS.

As mentioned in Section 3, C-MAPSS is a widely used public dataset generated using a turbofan engine simulation model called C-MAPSS (commercial modular aero-propulsion system simulation). This dataset simulates the degradation scenarios of turbofan engines under different operating conditions. Each dataset consists of a unit ID, cycle index, three values for the operational settings, and 21 time-series sensor measurements contaminated with noise [128]. Table 5 summarizes the available datasets, whose details can be found in references [129,130], and Figure 10 shows the diagram of C-MAPSS. To date, most of the research using the C-MAPSS datasets has been conducted by approach 1, in which the system is treated as a black-box, and focus is given to improving the RUL prediction accuracy by the machine learning model. However, the true value of system-level prognostics should be on the algorithm that can identify the faulty components and/or the fault modes and track their contribution to the system performance. To accomplish the true aim of system-level prognostics for the C-MAPSS dataset, not only the approach 1 (system health index) but also the approach 4 (multi failure modes) should be considered in the future to answer the following questions:

- Which fault mode of the system causes more degradation of the system?
- What is the relationship between component degradation and system performance?
- How can the failure thresholds be set for the components and system?

### 4.2. PHM Data Challenge 2018

**Figure 11:**
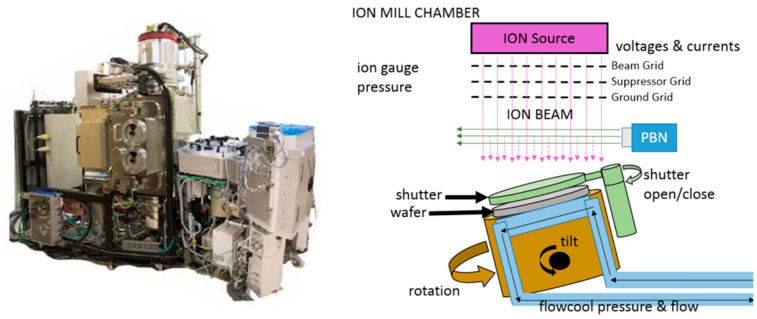
An Ion mill etching system and process.

In 2018, the dataset for the ion mill etch tool used in a wafer manufacturing process is published by the data challenge committee in the PHM society. In a wafer manufacturing process, the wafer is placed on a rotating fixture that is tilted at different angles. The wafer is shielded from the ion beam until it is ready for the milling process to begin using a shutter mechanism as shown in Figure 11. A Particle Beam Neutralizer (PBN) controls the ion beam as it travels to the wafer surface. In this process, the wafer is cooled by a helium/wafter system called flowcool. Many different types of failure mechanisms exist in this flowcool system. The objective is to build a model from time series sensors data collected from various ion mill etching tools operating under different conditions and settings. The model should diagnose the health state of the system and determine the RUL until the next failure of the system. The dataset corresponds to the 20 ion mill etch tools. Each dataset consists of 24 variables: 5 categorical variables, 14 numeric variables related to the operating conditions, and 5 sensor measurements. The committee mentioned that the system faces three different failure modes: ‘FlowCool Pressure Dropped Below Limit’, ‘Flowcool Pressure Too High Check Flowcool Pump’, and ‘Flowcool leak’. Different from the C-MAPSS data, these three faults do not correspond to the different subsystems or components of the system. It is unclear whether the three failure modes are interdependent or not since the dataset is obtained from a real industrial field. As a conclusion, approaches 1 (system health index), 3 (influenced components), and 4 (multi fault modes) should be considered for this problem to answer the following questions:

- How to obtain a degradation model from the datasets which face three different fault modes simultaneously?
- Which fault modes are interdependent or correlated?
- How to set the appropriate thresholds for the different fault modes?

## 5. Challenges for Practical System-Level Prognostics

**Figure 12:**
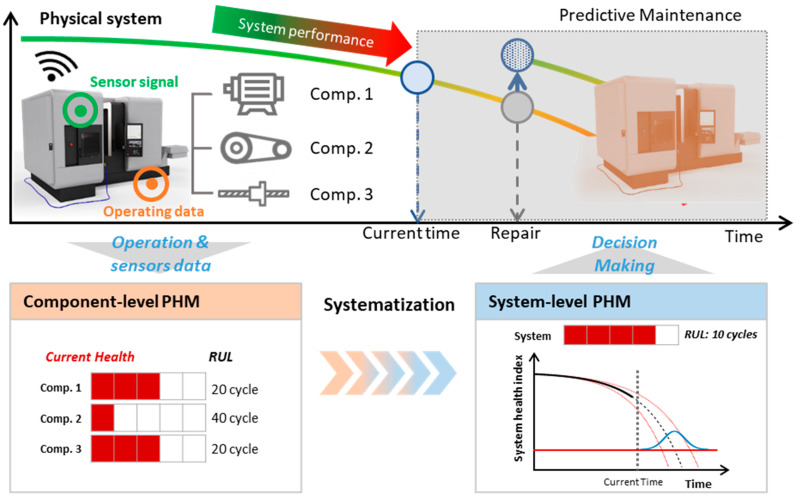
Illustration of system-level prognostics.

In Section 3, the current literature for system-level prognostics has been reviewed and grouped into four approaches, discussing their pros and cons. In this section, several challenges that should be overcome based on the review to accomplish the true aim of system-level prognostics are suggested. The challenges are divided into two categories: (1) systematization issues, and (2) general challenges. First, systematization issues arise from the definition of the system. Different from the component-level prognostics which typically consist of the three steps such as feature extraction, diagnostics, and prognostics, the system-level prognostics requires an additional step named ‘systematization’ as shown in Figure 12, which addresses the conversion of component-level information into the system-level. In fact, the abovementioned four approaches are more or less about how to perform ‘systematization’ in the system-level prognostics. Second is the general challenges, which refer to the issues that are not limited to but become more significant in the system-level prognostics, which is why this is addressed as a challenge of system-level prognostics.

### 5.1. Systematization Issues in System-Level Prognostics

In view of the systematization, the system-level prognostics has been classified into four approaches: system health index, component’s RUL, influenced components, and multi failure modes. For approach 1, system-level prognostics is conducted by analyzing the system health index. Depending on the types of CM data, existing literature has utilized either PSP or VSP methods. In the case of VSP, approach 1 benefits from its wide applicability since it does not require a high level of physical interpretation of the system. This means that approach 1 can be applied to a more complex system compared to the other three approaches. In practice, however, a large number of run-to-failure data for the CM and system degradation are needed to achieve satisfactory performance. Different from approach 1, approach 2 does not focus on extracting the system health index but integrates the component-level information to determine the system RUL based on the FTA. Approach 2 benefits for the situation where the system health index is not defined, or the system-level degradation model does not exist. However, domain knowledge and understanding of the system are required to build the appropriate standard for system failure. In approach 3, the interdependency between components’ degradations is exploited to make a more accurate RUL prediction. However, there is a practical difficulty in identifying the relationship between the component’s degradation. Therefore, the complexity will exponentially increase for the system with more than two or three components. Approach 3 will be suitable when the system consists of less than three components. Lastly, when the system or component has multiple failure modes, they are classified as approach 4. Different from the previous three approaches, this has been dealt with mainly in reliability engineering. Several algorithms have been derived from the reliability to apply to the prognostics. However, it is still very challenging to obtain the degradation pattern for different types of failure modes. Thus, approach 4 is appropriate when the system faces a failure of a particular component with multiple failure modes. As the approach becomes complicated, its applicability is limited to a simple system. It is important to select an appropriate approach considering the trade-off relationship between the level of complexity of the target system and approach. Table 6 summarizes the main characteristics of the four approaches with the titles A1~A4. Pros of each approach provide the opportunities of the system-level prognostics beyond the component-level prognostics. For cons, it describes the existing drawbacks of the listed approaches and suggests the challenges for each approach. Once the user defines the goal or the type of system-level prognostics, it is possible to utilize the existing algorithms or approaches.

### 5.2. General Challenges for System-Level Prognostics

#### 5.2.1. Big Data Management

As the sensor technology and the capacity of data storage are improved, the industry moves toward the era of big data, which enables engineers to develop PHM algorithms for complex systems more easily. Despite this advantage, however, there are still several issues to be explored in view of data management such as data storage and quality assessment. For instance, in the case of bearing prognostics, as the sampling rate of data acquisition becomes higher, the data size becomes bigger. Incessant data acquisition from the beginning to the end of life may not be a practical choice. To accomplish efficient data management, PHM designers should provide a practical standard for the time interval or amount of data suitable for the prognostic study. However, there is not enough literature on this subject. For example, Nguyen et al. [132] proposed a methodology for improving the inspection/monitoring policy to reduce the operation and maintenance costs but also ensure information quality. Jia et al. [133] introduced a method that assesses the data suitability for PHM based on detectability, diagnosability, and trendability which correspond to the performance of fault detection, diagnosis, and prognosis. More research is called for investigating this issue in big data situations for practical system prognostics.

#### 5.2.2. Prognostics under Data Deficiency

There is no doubt that components or systems are not allowed to run to failure in the field. Therefore, run-to-failure data are rare. As a result, it is desired to develop an RUL estimation approach when limited data are available. There are few publications that acknowledged this challenge and proposed approaches. Sobie et al. [134] introduced a simulation learning method that trains fault diagnostics algorithms with data which is generated by simulation from bearing dynamic models. Hu et al. [135] considered degradation data that reached predefined failure threshold as labeled data, whereas data without it as unlabeled ones. To utilize two different datasets, they proposed a co-training-based data-driven prognostic algorithm, denoted by COPROG, which uses two individual data-driven algorithms with each predicting RULs of censored units. Once the suspension units are labeled by a data-driven algorithm, another data-driven algorithm is trained by the training data labeled by the other. An et al. [136] demonstrated the method of utilizing accelerated life testing (ALT) degradation data for the prognostic of a system. Depending on the degradation model and loading conditions, four different ways of utilizing ALT data for prognostics are discussed. Kim et al. [137] proposed the data augmentation technique utilizing the run-to-fail (RTF) data obtained from different operating conditions. To predict the RUL under data deficiency, existing RTF data is mapped into the current operating condition and virtual RTF data sets are generated. Data deficiency is considered the major and basic obstacle to prognosis. Although there have been few publications, most of them were applied to component-level prognostics. As systems require higher safety operation and reliability, data deficiency becomes a more serious challenge at the system level. For this reason, data deficiency challenges should be overcome from components to systems.

#### 5.2.3. Online Performance Assessment and Correction

There are several prognostics metrics to evaluate the performance of prognostics algorithms, such as prognostic horizon (PH), α−λ performance, relative accuracy (RA), and convergence [138]. Traditional metrics focused on the offline analysis of prognostics algorithms using the run-to-failure data made in the past. In other words, these metrics are only available when the run-to-failure data exist. In practice, however, industrial systems are not allowed to operate until failure, and thus, it is difficult to employ the offline prognostics metric. Driven by this, the online performance assessment method is highly desired to evaluate the prognostics accuracy based on the current degradation trajectory. For this purpose, Hu et al. [139] proposed online metrics to evaluate the performance of model-based prognostics by monitoring only the current degradation trajectory without failure. Wang et al. [140] proposed a ranking method of PHM algorithms based on discrepancy without true failure data. As the system becomes more complex and requires higher safety operations, online performance assessment will be established as an essential tool for the application of prognosis.

#### 5.2.4. Uncertainty Management

The prediction of RUL is accomplished based on several prior steps, such as data collection, signal processing, feature extraction, and prognostics method selection. Each of these steps contains its own uncertainty, which propagates to the estimation of RUL. Uncertainty should be properly managed so that the uncertainty in RUL and the associated risk can be maintained below an acceptable level [141]. There are three main topics associated with uncertainty: (1) quantification, (2) propagation, and (3) management. Most of the existing research has focused on uncertainty quantification and propagation, which correspond to the process of identifying the various sources of uncertainty and combining them into the uncertainty in RUL prediction. System-level prognostics contains more uncertainty sources than component-level prognostics, such as uncertainties derived from multiple components or subsystems. Therefore, understanding how the uncertainty in a specific component propagates to the system quantifies the risk in system-level prognostics and allows system operators to determine which components should be repaired or inspected to obtain the desired system operating time. In addition, the system-level PHM process includes uncertainty from various functions, such as data acquisition, signal processing, fault diagnosis, and fault prognosis. Identifying these sources and their contribution to system-level RUL prediction can help PHM designers to obtain a final output with confidence by managing reducible uncertainties. For example, if acquired data shows an unacceptable level of uncertainty, maintenance engineers can update the sensor kit or increase the sampling rate to improve the quality of data [142].

#### 5.2.5. Strategy Transforming Scheduled Maintenance into Predictive Maintenance

In many industrial applications, the scheduled maintenance policy is already established. PHM is an important step to change the traditional scheduled maintenance policy for predictive maintenance. In practice, however, abrupt changes in maintenance policy can cause several side effects associated with safety and cost. Furthermore, the changes in maintenance strategy require approvals from various stakeholders such as manufacturers, maintenance operators, repair, and overhaul (MRO), and the federal aviation administration (FAA) [143]. Therefore, a systematic methodology for a gradual change from traditional scheduled maintenance to predictive maintenance is required.

## 6. Conclusions

In this paper, a review of the prognostics of multiple components systems, which are widely used in the industry, is provided. Different from component-level prognostics, system-level prognostics involves complex failure phenomena and interaction between components. To help understand this complexity, authors categorized existing approaches in the field of system-level prognostics into four groups: (1) health index-based approach, (2) components’ RUL integration, (3) influenced components, and (4) multiple failure modes. Each method has its own pros and cons, depending on available information and data. Engineers can choose the best method based on the available information and data of the specific system. Furthermore, general challenges that are not just limited to but significant to the system-level prognostics are summarized, hoping to inspire future research.

## Figures and Tables

**Table 1:** Summary of system health index-based approach.

Approach	System in the Study	Data Sources	Prognostics Algorithm
Physical System Performance	Water piping system	Direct CM	Dynamic reliability assessment [66]
	Pump system	Direct CM	Gamma process [64] Similarity-based method [78]
	Rectifier system	Direct CM	First-order reliability method (FORM) [31]
	Air conditioning system	Direct CM	Gamma process [64]
Virtual System Performance	Punching system	Direct CM	Bayesian network [79]
	Unmanned aerial vehicle system	Direct/Indirect CM data & environmental data	Bayesian network [65]
	Compressor system	Indirect CM data	Similarity-based method [80]
	Train door system	Indirect CM data	Generative adversarial network [81]
	Elevator door motion system	Indirect CM data	Autoregressive-moving average model [67]
	Aircraft engine (CMAPSS)	Indirect CM data	Similarity-based method [47,58,68] Particle filter [52,82] General path model [71] Ensemble of data-driven algorithm [77,83] Generative adversarial network [84]
Direct Remaining Useful Life	Aircraft engine (CMAPSS)	Indirect CM data	Multi-layer perceptron (MLP) [72,73] Recurrent neural network (RNN) [40,76] Long short-term memory (LSTM) [42,46,56] Convolutional neural network (CNN) [74,75]

**Table 2:** Summary of component RUL-based approach.

System in the Study	Algorithm	Characteristics
Aircraft ECS	Fault tree analysis & Kalman filter [85]	Fault tree-based RUL fusion Independent failure event
Aircraft hydraulic system	Fault tree analysis & Kalman filter [93]	Individual component’s RULs are estimated using Kalman filter and system-level RUL is determined based on Fault tree analysis
Electrical power system	Fault tree analysis [86,94]	Fault tree-based RUL fusion Optimum component combination to repair
	Kalman filter [95]	Individual component’s RUL is estimated using Kalman filter and defined as system-level RUL
Four-wheeled rover	Model decomposition [87]	Decomposition of a large prognostics problem into several Independent local subproblems
Pump	Model decomposition [88]	Novel distributed model-based prognostics scheme The system RUL is the minimum of all the distributed subsystem RULs
National Aerospace System	Model decomposition [89]	Combining individually independent components RULs of aircraft environmental control system
Centrifugal pump	Particle filter [90]	Individual component’s RULs are represented as particles and system-level RUL are approximated by them.
RF receiver system	Model decomposition [91]	Decomposing a system-level problem into multiple critical components
Numerical example	Petri net [92]	Incorporation of maintenance actions, various prognostics information, expert knowledge and resource availability

**Table 3:** Summary of prognostics of influenced components approach.

System in the Study	Algorithm	Characteristics
Tennessee Eastman Process	Inoperability input-output model [97,98,99,100,101,102]	Interaction between components Influence of the environment
Pump & Valve	Parallel Monte Carlo simulation &dynamic reliability assessment [103,110,111]	Interaction between components
Flue gas energy recovery system	Bayesian network [104]	Interaction between components Influence of the protection
Lorry system	Webuill model & Stochastic dependency model [106]	Interaction between components
Blast furnace wall	Multi-degradation modeling with public noise [107]	Interaction between components
Hydraulic hybrid system	Bond graph [112]	Interaction between components Dependency on operating mode
Gearbox	Marshall-Olkin bivariate exponential distribution [113]	Interaction between failure mode
Aircraft bleed system	System redundancy & Adaptation of operational modes in degraded functioning [105]	Interaction between components
Cold box unit in petrochemical plant	Regression [114]	Interaction between components
Numerical simulation	Structural impact measure [115] Stochastic modeling of interaction [108,109]	Interaction between components

**Table 4:** Summary of failure mode-based approach.

System in the Study	Algorithm	Types of Failure Mode
Rolling element bearing	Survival analysis [116]	Inner race fault Outer race fault Rolling element fault
	Particle filter [121]	Grease breakdown Spall Unknown fault
Pump system	Proportional hazard model [118]	Sealing ring wear Trust bearing damage
Electronic Throttle Control	Proportional hazard model [117,119]	Accelerator pedal Throttle Body Other three failure
Valve system	Particle filter [123]	Spring rate Internal leak Top (bottom) external leak Friction
Ion mill etching system (PHM Data challenge 2018)	Recurrent neural network (RNN) [124,125] Long short-term memory (LSTM) [126] Convolutional neural network (CNN) [127]	Flow pressure drop Flow pressure high Flow leakage

**Table 5:** C-MAPSS datasets [131].

Dataset	Training Data	Test Data	Operating Condition	Fault Mode
FD001	100	100	1	1 (HPC degradation)
FD002	260	259	6	1 (HPC degradation)
FD003	100	100	1	2 (HPC and Fan degradation)
FD004	249	248	6	2 (HPC and Fan degradation)

**Table 6:** Pros and cons of four system-level prognostics approaches.

Method	Pros	Cons
A1	Various types of prognostics algorithms can be applied once the one-dimensional performance is obtained. High level of system interpretation is not required.	Inappropriate classification of failure mode may reduce accuracy. Threshold setting for VSP can cause a wide uncertainty in RUL prediction.
A2	An algorithm can be developed from the component level. In the absence of a system-level degradation model, component-level RUL can be integrated to calculate the system RUL	Domain knowledge should exist to build the relationship between components and the system. All components are assumed to be independent.
A3	Component degradation that is accelerated by a linked component can be prevented.	It is challenging to model the dependency between degradations of components
A4	By monitoring potential failure modes of the system individually, this method can suggest which parts of the system should be repaired.	A large number of data and information is required corresponding to various failure modes.

## Data Availability

Not applicable.
